# Retrospective Study of Genetic Testing Results Reveals Pathogenic Variants Beyond BRCA1/2 in Hereditary Breast and Ovarian Cancer Cases in New Brunswick: Implications for Future Care

**DOI:** 10.1002/cam4.70640

**Published:** 2025-02-05

**Authors:** Kelly Gauvin, Véronique Allain, Nadia Bouhamdani, Chloe Williams, Yanis Saheb, Catherine Savoie, Lynn Macrae, Katherine Hodson, Yun Amber Zhu, Eric Allain, Mouna Ben Amor

**Affiliations:** ^1^ Université de Sherbrooke Sherbrooke Québec Canada; ^2^ Centre de Formation Médicale du Nouveau‐Brunswick Moncton New Brunswick Canada; ^3^ Vitalité Health Network Moncton New Brunswick Canada; ^4^ Department of Chemistry and Biochemistry Université de Moncton Moncton New Brunswick Canada; ^5^ Atlantic Cancer Research Institute Moncton New Brunswick Canada; ^6^ Genolife Saint‐Louis Québec Canada; ^7^ Dynacare Ontario Canada

## Abstract

**Background:**

In Canada, founder variants in breast cancer susceptibility genes have been identified in populations residing in Québec and Newfoundland, thus demonstrating the value in characterizing the genetic profile of local populations for better clinical management. New Brunswick has a diverse, yet genetically unexplored population that includes founder Irish and Acadian ancestry, among others, and we hypothesized that this population could demonstrate potential enrichments for variants in breast cancer genes.

**Methods:**

Health records were retrospectively analyzed for 445 cases referred to the genetics clinic in Moncton, New Brunswick, their molecular results were summarized and compared to allele frequencies from similar studies in Canada.

**Results:**

No ethnic or age‐related correlation for specific variants could be identified. However, *BRCA*1/2 variant frequency was lower than expected in the study group and variants in other susceptibility genes such as ATM and CHEK2 were higher when compared to similar studies.

**Perspectives:**

This study demonstrates a distinct profile in hereditary breast cancer genetics in a previously uncharacterized population, thus adding to existing knowledge of population genetics in Atlantic Canada.

## 1 Introduction

According to the Canadian Cancer Society, breast cancer (OMIM: 114480) is currently the most frequently diagnosed cancer type among Canadian women, with an estimation of 28,600 new cases in 2022, which represented 25% of all new cancer cases in women in the country. It was also estimated that 5500 women died from breast cancer in 2022, representing 14% of all cancer deaths in women. Ovarian cancer is less frequent with an estimation of 3000 new cases in 2022, which represented 2.7% of all new cancer cases in women. Overall, 1950 women were estimated to have died from ovarian cancer, which represent 5% of all cancer deaths in women [1]. Approximately 12% and 1.3% of the women will develop breast and ovarian cancer, respectively, at some point in their lives [2]. The province of New Brunswick (NB) is second behind Newfoundland and Labrador with the highest 25‐year tumor‐based prevalence followed by Nova Scotia. In fact, in 2023 it was estimated that there were 610 new cases of breast cancer and 65 new cases of ovarian, representing, respectively 23% and 2.5% of new cancer cases in women. Breast and ovarian cancer were thus responsible for 11% and 4.2% of cancer related deaths in women, respectively [1].

The identification of pathogenic variants in high‐ and moderate‐penetrance genes can greatly improve treatment strategies and surveillance [2]. The most well‐known genetic factors of hereditary breast and ovarian cancers are the high penetrance pathogenic variants in the *BRCA1* (HGNC:1100) and *BRCA2* (HGNC:1101) genes. The estimated prevalence of the *BRCA1* and *BRCA2* pathogenic variant carriers is up to 0.3% in the general population, 3% in women with breast cancer, 6% in women with early onset breast cancer, 10% in women with ovarian cancer, and 20% in high‐risk families [2, 3]. An estimated 10% of breast cancer cases are due to an underlying hereditary predisposition, such as a pathogenic/likely‐pathogenic variant in *BRCA1* or *BRCA2* [4, 5]. About 18% of all ovarian cancers are associated with *BRCA1/2* variants while another 6% are attributed to variants in other genes [6]. Women with germline *BRCA1* variants are estimated to have 60%–72% risk of breast cancer to age 70, while those with *BRCA2* variants are estimated to have 55%–88% risk. The ovarian cancer incidence by age 70 is 44%–59% in *BRCA1* carriers and 17%–35% in *BRCA2* carriers [7, 8, 9]. Additionally, several other genes have moderate penetrance for ovarian and breast cancers. In fact, a substantial fraction of *BRCA1/2* negative high‐risk families have been shown to harbor pathogenic variants in other genes, including *PALB2* (HGNC:26144), *CHEK2* (HGNC:16627), *ATM* (HGNC:795), and *TP53* (HGNC:11998). Furthermore, other families with a high likelihood of a mutation do not harbor a known pathogenic variant, demonstrating that much of the genetic contribution to breast cancer development has yet to be characterized; demonstrating the importance of multi‐gene panel testing beyond just *BRCA1/2* [10, 11, 12, 13].

Since 2018, a genetics service has been established in NB and oncogenetic evaluations have been offered at the Dr. Georges L.‐Dumont University Hospital Center in Moncton. The clinic currently follows the National Comprehensive Cancer Network Clinical Practice Guidelines in Oncology (NCCN guidelines) for the familial high‐risk assessment to determine participants' eligibility for genetic testing. However, we noticed that genetic testing results are uninformative for a large portion of tested participants, and we suspect that the panel of 24 genes used at our center covers only a fraction of our patients' needs and fail to identify a genetic cause for many NB breast or ovarian cancer patients.

The population of NB is genetically unique in several ways, including the founder Acadian and French‐Canadian populations. Both populations are already known to have a higher‐than‐average frequency of certain genetic disorders (e.g., Usher syndrome type IC [OMIM:276904] and Fanconi Reno tubular syndrome 5 Acadian variant [OMIM:618913]). Hence it is possible that genetic founder effects may exist for other disorders, including hereditary breast and ovarian cancer, such as in the French Canadian population of Quebec, which has been well studied [14, 15, 16]. However, the genetic aspects of hereditary breast and ovarian cancers specific to the NB population, have not yet been explored, making this an underserved population in Canada. In order to offer precise care tailored to our specific population within the province, it is opportune to evaluate the pertinence of the currently used gene panel for NB cancer patients to eventually see if there is a need to refine this targeted genetic screening panel.

## 2 Material and Methods

Genetic testing has been performed for each participant using a multi‐gene next generation sequencing panel of 24, 25, or 32 actionable genes (Supporting Information). Genetic testing was performed by a commercial laboratory (Fulgent Genetics or GeneDx). Briefly, genomic DNA was extracted from specimens and enriched for target coding regions using proprietary technology from GeneDx. Paired‐end sequencing was then carried out with Illumina sequencers. Reads were then aligned to GRCh37. Inclusion criteria were the following: have received a genetics consultation at the Medical Genetics Clinic between October 2019 and July 2022; have a personal and or a family history of breast or ovarian cancer; are eligible for genetic testing of hereditary breast and ovarian cancer based on the NCCN guidelines (Version 2.2022—Released March 9 2022); and have genetic test results available. Data were collected from electronic medical records (EMR) from 445 participants evaluated at our center. Summary descriptive statistics such as positivity rates were calculated and compared to reported incidence values in related literature. Data were analyzed using the tidyr (1.3.1), dplyr (1.1.4), and default packages for R v4.4.0.

## 3 Results

### 3.1 Sociodemographic Characteristics

**FIGURE 1:**
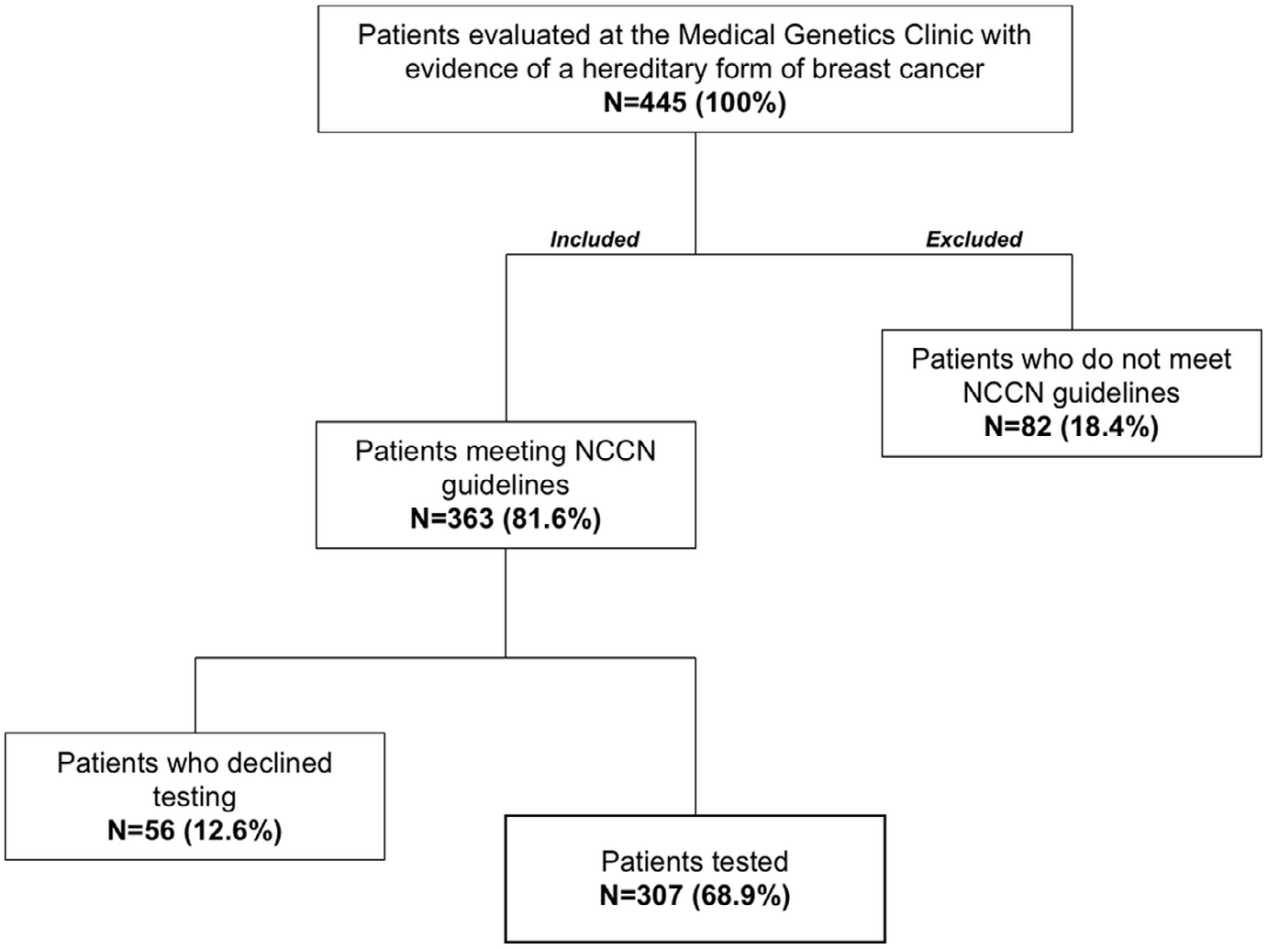
Overview of the patients included in the study group.

There was a total of 445 participants evaluated at our center for hereditary breast or ovarian cancer during the study period. Of these, 363 met NCCN testing criteria and 56 participants declined testing (Figure 1). The remaining 307 individuals had molecular testing results. Of these, 282 were females (92.2%) and 23 males (7.5%). Of the 307 participants that underwent molecular testing, 92 (30%) met mainstreaming criteria (see Supporting Information for details) as defined by our team, allowing oncologist, breast surgeons, and radio‐oncologists to order testing.

The age of cancer onset in our study group was under 50 years of age for 37.8% of participants and over 50 years of age for 39.4% of the study group (Table 1). NCCN criteria for genetic testing does not require that eligible individuals be affected by breast cancer if they have significant family history, therefore many participants included in our analysis have no data for age of cancer diagnosis.

**TABLE 1:** Sociodemographic characteristics of tested patients adhering to NCCN guidelines (*N* = 307).

Characteristics *N* (%)	
Sex at birth	
Female	283 (92.2)
Male	23 (7.5)
No data	1 (0.3)
Age at onset	
Under 50 years	116 (37.8)
Over 50 years	121 (39.4)
No data	70 (22.8)
Ethnicity^a^	
Acadian or French Canadians Origins^b^	119 (38.8)
European descent (non‐French or Acadian)	219 (71.3)
Others^b^	48 (15.6)
Metastatic disease	
Yes	23 (7.5)
No	186 (60.6)
Unknown	98 (31.9)
Laterality of breast cancer	
Bilateral	13 (4.2)
Unilateral	189 (61.6)
No data	105 (34.2)
Family history (first degree relative)	
Yes	62 (20.2)
No	244 (79.5)
No data	1 (0.3)
Cancer history	
Breast pathology	222 (72.3)
Ovarian pathology	7 (2.3)
Other pathology^c^	10 (3.2)
Unaffected	68 (22.1)

^a^ Ethnicity is self‐reported and does not add up to 100%.

^b^ Acadian and French Canadian includes all individuals who had the ‘Acadian’ or ‘French’ keywords in their medical records. Others category includes: South‐American, Native American, African or Asian origins.

^c^ Other pathology include: pancreatic, and prostate pathologies.

**FIGURE 2:**
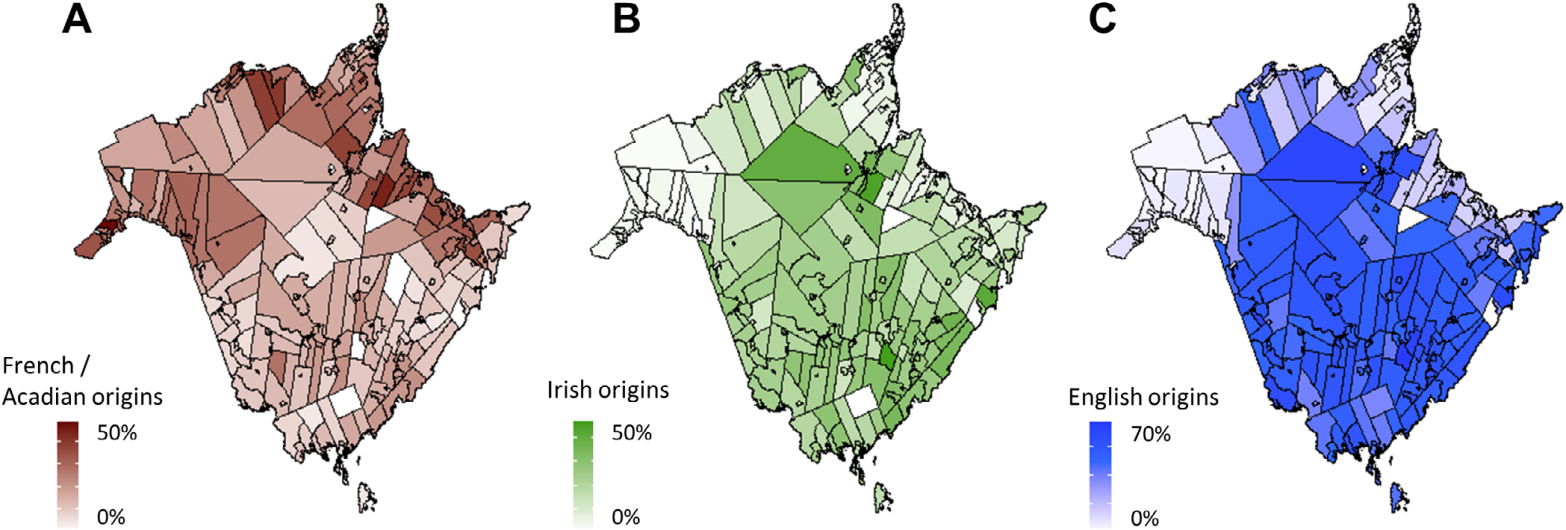
Geographical and ethnic stratification in New Brunswickers of European descent (A) Populations of French descendance along the eastern coast (B) Clusters of Irish descendance, especially in Saint‐John and Miramichi (C) other English origins in the rest of the province.

Participants were primarily of European descent (71.3%) and many had Acadian origins (38.8%), which is a major ethnic group in NB (Figure 2). Most had localized disease (60.6%), had unilateral breast cancer (61.6%), had no direct family history in first degree relatives (79.5%), and most were affected by breast cancer (72.3%, Table 1).

### 3.2 Breast Cancer Patients in NB Exhibit Few Recurring Variants, With Relatively Low BRCA1/BRCA2 Positivity Rates

Of the 307 participants with molecular results available, 268 underwent multi‐gene panel testing, whereas 39 underwent targeted predictive testing for a known familial variant. Of the panel tested individuals, 9.7% had either a pathogenic variant or a likely pathogenic (P/LP) variant, 22.8% had a variant of uncertain significance, and 67.5% had a negative result (Table 2). From the 26 P/LP results, 80.8% had a pathogenic variant only and 19.2% had both a pathogenic and a variant of uncertain significance. With 39 predictive tests, 12 P/LP results were obtained (30.8%). In total, 12.4% of participants have at least one pathogenic variant, and 4.9% had more than one variant. There were 102 unique variants in 26 genes.

**TABLE 2:** Panel and predictive testing results of patients adhering to NCCN guidelines.

	Panel testing (*N* = 268)	Predictive testing (P/LP) (*N* = 39)
Positive result	26	12
Pathogenic and VUS	5	—
Pathogenic only	21	—
VUS only	61	—
Negative result	181	27

**FIGURE 3:**
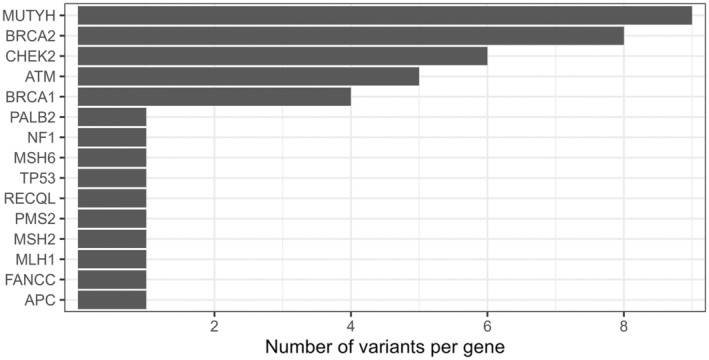
Total number of variants per gene in the study population only including pathogenic or likely pathogenic variants.

Variants in *BRCA2*, *ATM*, *CHEK2*, *BRCA1 and MUTYH* (HGNC:7527) were the most frequent P/LP variants. *PALB2*, *FANCM* (HGNC:23168), *ATM*, *BRCA1* and *CHEK2* also had several VUS (Figure 3; Table S1). In summary 31.6% of participants with a P/LP result had variants in *BRCA1*/*2*, 15.8% had a *CHEK2* variant, while *ATM* variants represented 13.2% of all P/LP results.

The most recurring variant was the *MUTYH* gene variant c.1187G>A (p.Gly396Asp) present in 6 tested individuals (1.95%). Besides the *CHEK2* variant c.1100delC (p.Thr367MetfsX15) identified in three cases, the other variants were found in either only two participants or unique cases. No enrichment of variants or higher positivity rate could be detected in either age or ethnic group.

## 4 Discussion

The information gathered on demographics concords with those of a study conducted in Ontario in 2020 on multi‐gene panel testing for hereditary breast and ovarian cancer [17]. In our study, males represented 7.5% of the cohort. The cohort was comprised of similar number of participants that had an early onset diagnosis of cancer (under 50 years old) compared to a late onset (50 years old and over) of breast or ovarian cancer.

Despite ethnicity not being an appropriate proxy for genetic ancestry, we explored if there might be any trends, enrichments, or correlations within specific groups. However, it was difficult to determine if any genetic variants were enriched in a specific ethnic group due to the fact that ethnic information was self‐reported by each patient during clinical genetic sessions. Consequently, participants could self‐identify as multiple ethnicities, thus making stratification of participants on this basis complex. Regardless, and despite clear links with founder populations in Québec, our analysis showed no enrichment even in those self‐reporting as having Acadian or French ancestry, the second largest ethnic subgroup in NB.

In most of the participants where multi‐gene panel genetic testing identified a variant; the result was a variant of uncertain significance. This is in line with the findings of other similar studies [11, 17]. Such results pose significant challenges for patient care plans, as it is often unclear how to proceed with these findings. Furthermore, many variants of uncertain significance are reclassified at a later time. This was the case for three of the cases in this study, one of whom was initially found to have a VUS but was later reclassified as a likely pathogenic variant and two who were reclassified from VUS to likely benign. It is of utmost importance to study and reevaluate these variants to be able to better understand the impact they may have on the patient and to develop a standardized care plan.

The P/LP variant rates in our cohort were slightly different than studies conducted in Ontario and Québec [14, 15, 17, 18]. We found that the participants in our region have an unusually high *ATM* frequency [10, 11, 17]. To our knowledge, there is no data or medical registry on the regional incidence of Ataxia Telangiectasia or hereditary ATM‐related cancer, however if this higher rate of ATM variation in NB is validated, it may have significant implications for breast cancer management in our population. Patients with germline pathogenic ATM variants have increased radiosensitivity and this may lead to very severe complications [19]. Thus, molecular results may help tailor management of breast cancer and the option of preconception screening for Ataxia Telangiectasia in patient's spouses. Despite the elevated *ATM* frequency result, *MUTYH* and *BRCA2* remained the most frequently observed genes with pathogenic or likely pathogenic variants, at 23.7% and 21.1% of cases with P/LP variants, respectively. In regard to *BRCA1*/*2* variants, they represent 31.6% of cases with P/LP variants. This differs from the above‐mentioned studies which have shown that *BRCA1/2* represented over 50% of their P/LP variants [10, 11, 17].

Three monoallelic *MUTYH* variants accounted for 23.7% of P/LP results (rs36053993, rs587780751 and rs34612342). This is likely an incidental finding and not related to the reason for testing. Monoallelic carriers of *MUTYH* pathogenic variants are not at increased risk for cancer, and it is known that roughly 1%–2% of people of European ancestry carry a monoallelic *MUTYH* pathogenic variant [20]. Two recurrent pathogenic variants (c.536A>G, p.Y179C and c.1187G>A, p.G396D) account for between 50% and 82% of the pathogenic variants in the white European population [21]. The CHEK2*1100delC variant (rs555607708) was also detected in our study group. This variant, which is associated with a 3‐ to 5‐fold increase in risk of breast cancer, is also prevalent in people of European ancestry [22, 23].

No discernable recurrent variations were observed, even when stratified by ethnic or age group. It is difficult to comment on the lack of recurrent variants in our study group, and we may not be able to apply this to the NB population at large, given the limitations of our small sample size. Notably, comparable studies in French Canadian populations focused on one particular ethnicity, and inclusion criteria in these studies are strict in terms of reporting French Canadian ancestry, compared to our group which can self‐report ancestry with no restrictions for inclusion [14, 15, 18]. Four of the previously described founder variants in French Canadians were not found to be enriched in our study cohort. This includes the *BRCA1* variants c.4327C>T (rs41293455), which was only measured in one participant from this study, and c.2834_2836delinsC (rs386134270), which was undetected. As for *BRCA2*, the c.8537_8538del variant (rs80359714) was detected once and the c.5857G>T variant (rs80358814) was not observed. These were the four most reported variants in a study by Tonin and colleagues [15]. We cannot be certain that the NB population has a similar genetic profile to the French Canadian population from Quebec, but this study suggests that these founder variants may not be present at very high rates in NB.

Limitations of this study include a small sample size (445 participants) which can be attributed to the location of the clinic within NB, a province where the population is distributed across a large geographical area. Other regions of the province may have different ethnic compositions as shown in Figure 2. To orient future research, a better capture of the other regions of the province could be done through multi‐center sampling to ensure a more accurate representation of the NB population as a whole. To date, there has only been one other multi‐gene study in Atlantic Canada examining hereditary breast cancer in Newfoundland and Labrador, where a *RAD51C* (HGNC:9820) variant (rs587780257) was found at a higher allele frequency than in reference populations and showed an autosomal dominant, low‐penetrance inheritance pattern [24]. However, this variant was not found in our study group. A second study performed with participants residing in Newfoundland and Labrador also reported numerous *BRCA1/2* variants, but our study group had only two variants in common with this cohort, neither of which were particularly enriched in either population [25].

Another limiting factor in our study was missing information from the participants' charts. This was especially a challenge regarding the HER2 status for those with breast cancer, as well as the presence or absence of metastatic disease. This has impacted our ability to correlate these parameters with the variants that have been identified in participants.

## 5 Conclusion

To our knowledge this is the first study examining the landscape of heritable genetic variants associated with breast cancer in NB. It provides new data describing the unique risks and genetic makeup of the population in this region of Canada. A multi‐center study including other regions of NB and neighboring provinces would provide a clearer picture of variant frequencies in Atlantic Canada. Therefore, more data is needed to support our findings that *BRCA1/2* incidence is lower in NB, giving way to higher frequencies of variants in other breast cancer genes such as *ATM*.

## Author Contributions

**Kelly Gauvin:** data curation, writing – review and editing, writing – original draft, formal analysis. **Véronique Allain:** data curation, writing – original draft, formal analysis, writing – review and editing. **Nadia Bouhamdani:** formal analysis, writing – original draft, writing – review and editing, visualization. **Chloe Williams:** data curation, writing – review and editing, visualization, formal analysis. **Yanis Saheb:** data curation, writing – review and editing. **Catherine Savoie:** data curation, writing – review and editing. **Lynn Macrae:** validation, writing – review and editing. **Katherine Hodson:** writing – review and editing, formal analysis, data curation, validation, writing – original draft. **Yun Amber Zhu:** writing – review and editing, formal analysis, data curation. **Eric Allain:** supervision, formal analysis, data curation, visualization, writing – review and editing, writing – original draft, funding acquisition, investigation, project administration. **Mouna Ben Amor:** conceptualization, funding acquisition, writing – review and editing, supervision, formal analysis, project administration.

## Ethics Statement

This study was approved by the Vitalité Health Network Research Ethics Board (#101703). No informed consent was gathered given the retrospective design of the study.

## Conflicts of Interest

The authors declare no conflicts of interest.

## Supporting information


Data S1.

## Data Availability

Data is available upon request.
