# Supplementary material for: Retrospective Study of Genetic Testing Results Reveals Pathogenic Variants Beyond BRCA1/2 in Hereditary Breast and Ovarian Cancer Cases in New Brunswick: Implications for Future Care
Source: Cancer Med. 2025 Feb 5;14(3):e70640. doi: 10.1002/cam4.70640 (PMC11795414; doi:10.1002/cam4.70640)
Supplement: Supplementary file 1 — Data S1. [file CAM4-14-e70640-s001.docx]

**Breast cancer patients: Criteria for mainstream genetic testing**

Patients who have been diagnosed with breast cancer recently and meet any of the following criteria.

1. Treatment implications:
   - To aid in systemic treatment decisions using PARP inhibitors for breast cancer in the metastatic setting
   - To aid in adjuvant treatment decisions with Olaparib for high-risk, HER2-negative breast cancer
2. Personal history of breast cancer ≤ 50 years old
3. Breast cancer with ovarian or pancreatic cancer
4. Male breast cancer
5. Triple-negative breast cancer
6. Multiple primary breast cancers (synchronous or metachronous)

**Multi-gene sequencing panel information**

Fulgent 32 gene panel: *ATM, BARD1, BRCA1, BRCA2, BRIP1, CDH1, CHEK2, EPCAM, FANCC, FANCM, MLH1, MRE11, MSH2, MSH6, MUTYH, NBN, NF1, NTHL1, PALB2, PIK3CA, PMS2, PTEN, RAD50, RAD51C, RAD51D, RECQL, SDHB, SDHD, SMARCA4, STK11, TP53, XRCC2*

GeneDx 25 gene panel:*ATM, BARD1, BRCA1, BRCA2, BRIP1, CDH1, CHEK2, EPCAM, FANCC, FANCM, MLH1, MSH2, MSH6, MUTYH, NB1, NF1, PALB2, PMS2, POLD1, PTEN, RAD51C, RAD51D, RECQL, STK11, TP53*

GeneDx 24 gene panel: *ATM, BARD1, BRCA1, BRCA2, BRIP1, CDH1, CHEK2, EPCAM, FANCC, FANCM, MLH1, MSH2, MSH6, MUTYH, NF1, PALB2, PMS2, POLD1, PTEN, RAD51C, RAD51D, RECQL, STK11, TP53*

**Table S1: Type and number of genetic variants identified in our cohort**

| GENE NAME | P/LP/VUS | VARIANT (HGVS compliant where possible*) | Number of variants |
| --- | --- | --- | --- |
| *APC* | VUS | NM_000038.6:c.854A>G | 2 |
|  | P | NM_000038.6:c.3920T>A | 1 |
| *ATM* | VUS | NM_000051.4:c.7358G>A | 1 |
|  | VUS | NM_000051.4:c.2149C>T | 1 |
|  | VUS | NM_000051.4:c.3299C>T | 1 |
|  | VUS | NM_000051.4:c.3402+3A>G | 1 |
|  | VUS | NM_000051.4:c.3577G>A | 1 |
|  | VUS | NM_000051.4:c.3689A>G | *1* |
|  | VUS | NM_000051.4:c.5566G>C | 1 |
|  | VUS | NM_000051.4:c.5821G>C | 1 |
|  | VUS | NM_000051.4:c.6537T>G | 1 |
|  | LP | NM_000051.4:c.5675-7_5675-4del | 1 |
|  | P | NM_000051.4:c.7271T>G | 1 |
|  | P | NM_000051.4:c.2251-10T>G | 1 |
|  | P | NM_000051.4:c.7388_7389insAlu* | 2 |
|  | VUS | NM_000051.4:c.6543G>T | 1 |
| *AXIN2* | VUS | NM_004655.4:c.544A>G | 1 |
| *BARD1* | VUS | NM_000465.4:c.1402G>A | 1 |
|  | VUS | NM_000465.4:c.1810+3G>T | 1 |
|  | VUS | NM_000465.4:c.2315T>A | 2 |
| BRCA1 | VUS | NM_007294.4:c.914G>A | 1 |
|  | VUS | NM_007294.4:c.301+1G>T | 1 |
|  | VUS | NM_007294.4:c.4144T>A | 1 |
|  | VUS | NM_007294.4:c.4903G>A | 1 |
|  | VUS | NM_007294.4:c.670G>A | 1 |
|  | P | NM_007294.4:c.4327C>T | 1 |
|  | P | NM_007294.4:c.2125_2126insA | 2 |
|  | P | NM_007294.4:c.5266dup | 1 |
| *BRCA2* | VUS | NM_000059.4:c.694T>C | 1 |
|  | VUS | NM_000059.4:c.5390C>G | 1 |
|  | P | NM_000059.4:c.158_159insAlu* | 1 |
|  | P | NM_000059.4:c.5909C>A | 2 |
|  | P | NM_000059.4:c.6206T>G | 1 |
|  | P | NM_000059.4:c.8537_8538del | 1 |
|  | P | NM_000059.4:c.9117G>A | 1 |
|  | P | NM_000059.4:c.9435_9436del | 2 |
| *CHEK2* | VUS | NM_007194.4:c.1078G>A | 1 |
|  | VUS | NM_007194.4:c.1460A>G | 1 |
|  | VUS | NM_007194.4:c.14C>T | 1 |
|  | VUS | NM_007194.4:c.661A>G | 1 |
|  | VUS | NM_007194.4:c.715G>A | 1 |
|  | VUS | Duplication Exons 3-15* | 1 |
|  | LP | NM_007194.4:c.433C>T | 1 |
|  | LP | Deletion exon 14* | 1 |
|  | P | NM_007194.4:c.1100del | 3 |
|  | P | NM_007194.4:c.283C>T | 1 |
| *FANCC* | VUS | NM_000136.3:c.1607T>C | 1 |
|  | VUS | NM_000136.3:c.176C>T | 1 |
|  | VUS | NM_000136.3:c.28T>G | 1 |
|  | LP | NM_000136.3:c.843+1G>A | 1 |
| *FANCM* | VUS | NM_020937.4:c.1576C>G | 1 |
|  | VUS | NM_020937.4:c.2452A>G | 1 |
|  | VUS | NM_020937.4:c.4622C>T | 1 |
|  | VUS | NM_020937.4:c.5848T>G | 1 |
|  | VUS | NM_020937.4:c.874C>G | 1 |
| *MLH1* | VUS | NM_000249.4:c.2024G>T | 1 |
|  | P | NM_000249.4:c.1713del | 1 |
| *MSH2* | VUS | NM_000251.3:c.1804C>G | 2 |
|  | VUS | NM_000251.3:c.2517T>A | 1 |
|  | VUS | NM_000251.3:c.74G>A | 1 |
|  | P | NM_000251.3:c.942+3A>T | 1 |
| *MSH6* | VUS | NM_000179.3:c.193T>G | 1 |
|  | VUS | NM_000179.3:c.3388G>A | 1 |
|  | VUS | NM_000179.3:c.3509T>C | 1 |
|  | P | NM_000179.3:c.10C>T | 1 |
| *MUTYH* | VUS | NM_001128425.1:c.1393C>G | 1 |
|  | P | NM_001128425.1:c.1187G>A | 6 |
|  | P | NM_001128425.1:c.536A>G | 1 |
|  | P | NM_001128425.1:c.933+3A>C | 2 |
| *NBN* | VUS | NM_002485.5:c.1113A>T | 1 |
|  | VUS | NM_002485.5:c.83G>A | 1 |
| *NF1* | VUS | NM_000267.3:c.1553C>T | 1 |
|  | VUS | NM_000267.3:c.4164G>A | 1 |
|  | VUS | NM_000267.3:c.6985A>C | 1 |
|  | LP | NM_000267.3:c.4972_4973dup | 1 |
| *NTHL1* | VUS | NM_002528.5:c.527T>C | 1 |
| *PALB2* | VUS | NM_024675.4:c.13C>T | 1 |
|  | VUS | NM_024675.4:c.1647C>A | 1 |
|  | VUS | NM_024675.4:c.2773G>C | 1 |
|  | VUS | NM_024675.4:c.3146T>C | 1 |
|  | VUS | NM_024675.4:c.94C>G | 1 |
|  | P | NM_024675.4:c.599del | 1 |
| *PMS2* | VUS | NM_000535.7:c.1399G>A | 1 |
|  | VUS | NM_000535.7:c.354-5C>G | 2 |
| *POLD1* | VUS | NM_002691.4:c.233G>A | 1 |
|  | VUS | NM_002691.4:c.2794G>C | 1 |
|  | VUS | NM_002691.4:c.766G>C | 1 |
| *POLE* | VUS | NM_006231.4:c.2683G>A | 1 |
|  | VUS | NM_006231.4:c.4168C>T | 1 |
| *RAD51C* | VUS | NM_058216.3:c.190A>T | 1 |
| *RAD51D* | VUS | NM_002878.4:c.26G>C | 2 |
|  | VUS | NM_002878.4:c.422T>C | 1 |
| *RECQL* | VUS | NM_002907.4:c.1798-2A>C | 1 |
|  | VUS | NM_002907.4:c.2T>C | 1 |
|  | VUS | NM_002907.4:c.325G>A | 1 |
|  | P | NM_002907.4:c.1667_1667+3del | 1 |
| *SMAD4* | VUS | NM_005359.6:c.535A>G | 1 |
| *STK11* | VUS | NM_000455.5:c.1189G>T | 1 |
|  | VUS | NM_000455.5:c.1228G>A | 1 |
| *TP53* | P | NM_000546.6:c.207delinsCTCTG | 1 |

*Large deletions and Alu insertions where the length or sequence could not be resolved due to limitations in the technology could not be validated to be HGVS compliant.

**Table S2: Demographics information broken down by biological sex.**

| Age at Onset | Females | Males |
| --- | --- | --- |
| *Under 50 years* | 113 | 3 |
| *Over 50 years* | 114 | 6 |
| *No Data* | 56 | 14 |
| Ethnicity^1^ |  |  |
| *Acadian or French Canadians Origins^2^* | 110 | 9 |
| *European Descent (non-French or Acadian)* | 209 | 10 |
| *Others^2^* | 44 | 4 |
| Metastatic Disease |  |  |
| *Yes* | 23 | 0 |
| *No* | 178 | 8 |
| Unknown | 83 | 15 |
| Laterality of Breast Cancer |  |  |
| *Bilateral* | 13 | 0 |
| *Unilateral* | 185 | 4 |
| *No Data* | 86 | 19 |
| Family History (1^st^ degree relative) |  |  |
| *Yes* | 55 | 7 |
| *No* | 228 | 16 |
| *No Data* | 1 | 0 |
| Cancer History |  |  |
| *Breast pathology* | 217 | 5 |
| *Ovarian pathology* | 7 | 0 |
| *Other Pathology^3^* | 10 | 0 |
| *Unaffected* | 50 | 18 |

1. Ethnicity is self-reported and does not add up to 100%.
2. Acadian and French Canadian includes all individuals who had the ‘Acadian’ or ‘French’ keywords in their medical records. Others category includes: South-American, Native American, African or Asian origins
3. Other pathology include: pancreatic, and prostate pathologies
